# Implementation of Robotic-Assisted Sacrocervicopexy for Apical Organ Prolapse Using the Semitendinosus Tendon—Pilot Study and Analysis of Clinical Outcome

**DOI:** 10.1007/s00192-024-05975-1

**Published:** 2024-11-12

**Authors:** Carolin Schröder, Charlotte Lukannek, Eva K. Egger, Lucia A. Otten, Alexander Mustea, Dominique Koensgen

**Affiliations:** Department of Gynecology and Gynecological Oncology, University Hospital Bonn, Venusberg Campus 1, 53127 Bonn, Germany

**Keywords:** Apical pelvic organ prolapse, Semitendinosus tendon, Da Vinci robotic system

## Abstract

**Introduction and Hypothesis:**

This video demonstrates a robotic-assisted sacrocervicopexy using the semitendinosus tendon.

**Methods:**

Between June 2022 and February 2023, we performed the worldwide first Da Vinci robotic-assisted sacrocervicopexies (SCP) for apical organ prolapse using the semitendinosus tendon of the left knee. Analysis of safety, feasibility, and clinical outcome of the first ten patients operated on using this new surgical technique included the German pelvic floor questionnaire (GPFQ) as well as a clinical examination.

**Results:**

Ten patients with a follow-up of 12 months were included. There was a significant reduction of the patient’s symptoms according to the GPFQ regarding the domain bladder (preoperatively versus 3 months postoperatively, mean 3.85 vs 1.61, *p* = 0.034), total score (preoperatively versus 3 months postoperatively, mean 12.79 vs 3.28, *p* = 0.034), and descensus symptoms (preoperatively versus 12 months postoperatively, mean 4.74 vs 0.67, *p* = 0.022). POP-Q stage (point C) was significantly reduced between the preoperative period and at the time of discharge (mean 2.2 vs 0, *p* = 0.004). No serious intra- and postoperative complications occurred.

**Conclusions:**

This pilot study showed satisfying clinical outcomes after a follow-up of 12 months, with a low mid-term complication rate.

**Supplementary Information:**

The online version contains supplementary material available at 10.1007/s00192-024-05975-1.

## Introduction

Laparoscopic sacrocervicopexy (SCP) is a successful surgical procedure for apical prolapse repair, with low complication rates [1]. Native tissue vaginal repairs have the lowest complication rate, but have a higher risk of recurrence [2]. Semitendinosus tendons are the gold standard in orthopedic surgery, with a regeneration rate of up to 72% 2 years after harvesting [3, 4]. A pilot study by Hornemann et al. showed promising success rates using the semitendinosus tendon for SCP, verified in a larger study in 2023 including 113 patients [5, 6]. To our knowledge, the robotic approach of SCP using the semitendinosus tendon has not yet been described.

With a 12-month follow-up, this pilot study was aimed at evaluating the feasibility and safety of the implementation of the first robotic-assisted SCP worldwide.

## Materials and Methods

The Department of Urogynecology at University Hospital Bonn, Germany, is the first clinic worldwide to perform Da Vinci robotic-assisted SCP using the tendon of the semitendinosus muscle. This pilot study analyzed the 1-year clinical outcome of the first ten patients, who underwent the procedure after conservative treatment was unsuccessful or not accepted. The hypothesis of our study was that robotic-assisted SCP is a feasible approach resulting in a reduction of POP-Q point C, with a low complication rate. The primary outcome was the change in POP-Q from baseline to the 12-month follow-up, whereas secondary outcomes included changes in the German Pelvic Floor Questionnaire (GPFQ) scores (bladder and bowel function, prolapse, sexuality) and intra- and postoperative complications [7]. All patients were operated on by the same surgeon. Gynecological examination was performed when the patient’s bladder was empty.

Patients with apical organ prolapse (POP-Q point C ≥ −1) and normal Pap smear, taken as part of regular screening, were included after conservative treatment was unsuccessful or not accepted. All patients provided informed consent.

Patients with a POP-Q point C of < −1 or without a normal PAP smear were excluded.

### Statistical Analysis

Statistical analysis was performed using SPSS 29.0 (IBM Corp. Released 2022 IBM SPSS Statistics for Windows, Version 29.0; IBM, Armonk, NY, USA). Descriptive analysis was performed and an explorative analysis using the Friedman test with post hoc signed-rank test was conducted for ordinal values comparing preoperative values with those at the time at discharge, and 3 and 12 months after the operation. There was no imputation for missing values. A *p* value of 0.05 was seen as statistically significant. Owing to the retrospective design, no power calculation was done. The need for ethics approval and consent was waived.

### Video: Implementation of the Robotic-Assisted Sacrocervicopexy Using the Semitendinosus Tendon

The surgery is performed under general anesthesia. The semitendinosus tendon is stripped from the popliteal fossa of the patient’s left leg. A 25-mm incision is made and the fascia is incised over the semitendinosus tendon (video minutes 00:00 to 00:50). Then, an open 7-mm tendon harvester (Arthrex, Naples, FL, USA) is put over the tendon (video minutes 00:50). By pushing it proximally, this part of the tendon is separated from the semitendinosus muscle (video minutes 00:52 to 01:13). The distal part of the tendon is removed from the pes anserinus with a 7-mm closed harvester (Arthrex) in the same manner (video minutes 01:25 to 01:42). The incision in the popliteal cavity is closed with two single stitches. Both ends of the removed tendon are prepared using a sling suture (HoTT®-Sling, Hornemann Tendon Transplantation, absorbable, set 2 + 1; SMI, St. Vith, Belgium, for SCP, and non-absorbable, set 3; SMI, for sacrocolpopexy) in order to prevent the splitting of the tendon (video minutes 02:44 to 03:40). From the fourth operation on, we sutured a straight needle to the tendon to improve the intra-abdominal handling of the tendon (video minutes 03:41 to 04:07).

Three robotic trocars are placed umbilically and 8 cm lateral to each side. An additional 10-mm assist trocar is placed 8 cm lateral to the right robotic trocar. Next, a laparoscopic supracervical hysterectomy is performed (± salpingectomy or adnexectomy depending on menopausal status; not shown in the video). Then, the cervix is prepared. Therefore, the vesico-uterine pouch is opened and the bladder is moved apart from the cervix (video minutes 04:20 to 04:35). The peritoneum is then tunneled on the right side of the pelvis (video minute 04:36).

During the first operation, two perforations with a monopolar spatula (CleanCoat™ Laparoscopic Curved 36 cm, Covidien™) were made through the cervix in a ventral to dorsal-cranial direction. Therefore, extra suprasymphyseal access was used. From the second operation on, there was only one perforation of the cervix, with no extra suprasymphyseal access, as the wide mobility of the robot arms was fully utilized (video minutes 04:47 to 05:00). The prepared tendon is introduced into the abdomen via the 10-mm assist trocar. The tendon is pulled through the tunnel of the cervix using the straight needle sutured temporarily to the tendon (video minutes 05:01 to 05:21).

A fenestrated bipolar clamp and a monopolar scissor are used to dissect the anterior longitudinal ligament with a nerve-sparing technique. It is then incised and tunneled in the region of the promontory (L5/S1) over a length of 20 mm (video minutes 05:22 to 05:48). The tendon is then passed through the peritoneal tunnel and pulled through the tunnel of the anterior longitudinal ligament (video minutes 05:49 to 06:08). The ends of the tendon get attached by a knot and a side-to-side sling suture (video minutes 06:09 to 06:45).

From the tenth operation on, we added a suture of the tendon to the cervix using two Ethibond sutures to prevent the rupture of the prepared tunnel of the cervix (video minutes 06:46 to 07:13). The peritoneum is closed with resorbable sutures (video minutes 07:13 to 07:33).

## Results

Between June 2022 and February 2023, ten patients were operated on. The median age was 55 years (range 44 to 70, SD 8.8), the median BMI was 26 kg/m^2^ (range 21–29, SD 2.4). Seven patients had a POP-Q stage II of point C (70%), three patients had stage III of POP-Q point C (30%). None of our patients had a recurrent apical prolapse.

Table 1 shows additional procedures that were performed as well as intra- and postoperative complications. As shown in Table 2, all patients had POP-Q point C between −8 and −7 at the time of discharge. There was a significant reduction in the POP-Q stages between the preoperative period and the time of discharge (mean 2.2 vs 0, *p* = 0.004). Three months after surgery, three patients had a cystocele and/or rectocele grade I, which were treated conservatively. Two patients were stable after 12 months and one developed a stage II rectocele. One patient complained of pain when moving the left leg and received physiotherapy (Table 2).

**Table 1:** Intra- and perioperative characteristics

Characteristic	Data
Procedures	
Sacrocervicopexy, LASH, *n* (%)	9 (90)
Sacrocolpopexy	1 (10)^a^
Anterior/posterior colporrhaphy	10 (100)
Burch colposuspension	4 (40)
Complications, *n* (%)	
Intraoperative	0 (0)
Postoperative	1 (10)^b^
Hospital stay (days), mean, range (SD)	5, 4–7, (0.7)

*LASH* laparoscopic supracervical hysterectomy including salpingectomy for premenopausal and adnexectomy for postmenopausal patients

^a^Previous hysterectomy

^b^Abdominal wall hematoma

**Table 2:** Summary of Pelvic Organ Prolapse Quantification (POP-Q) stage

POP-Q stage	Preoperative POP-Q point C, *n* (%)	At discharge, *n* (%)	*p* value*, *n*	3 months after operation, *n* (%)	12 months after operation, *n* (%)
0	0 (0)	10 (100)^a^		7 (70)^a^	6 (60)^a^
1	0 (0)	0 (0)		3 (30)^b^	2 (20)^c^
2	8 (80)	0 (0)		0	1 (10)^d^
3	2 (20)	0 (0)		0	0
4	0 (0)	0 (0)		0	0
N.A	0 (0)	0 (0)		0	1 (10)
Total, mean, median (SD)	2.2, 2 (0.42)	0, 0 (0)	0.004*, (*n* = 7)	0.3, 0 (0.48)	0.44, 0 (0.73)

*NA* not available

*Friedman test with post hoc signed-rank test, *n* = number of included patients for analysis without imputation

^a^All three levels (and POP-Q C between −8 and −7)

^b^Two patients with cystocele grade I (POP-Q Ba = −2), one patient with rectocele grade I (POP-Q Bp = −2)

^c^Two patients with cystocele grade I (POP-Q Ba = −2)

^d^One patient with rectocele grade II (POP-Q Ba = 0)

Three months postoperatively, there was a significant reduction in the mean score regarding bladder function and total score in the GPFQ (3.85 vs 1.61, *p* = 0.034 and 12.79 vs 3.28, *p* = 0.034). The prolapse score also showed a reduction between the preoperative period and 3 months postoperatively, but this was only significant between the preoperative period and 12 months postoperatively (4.74 vs 0.86 vs 0.67, *p* = 0.022; Table 3).

**Table 3:** German Pelvic Floor Questionnaire results

Domains	Preoperatively, mean (SD)	3 months after operation, mean (SD)	*p* value* (*n*)	12 months after operation, mean (SD)	*p* value*
Bladder function	3.85 (2.1)	1.61 (1.13)	0.034 (*n* = 5)	1.37 (0.92)	0.081
Bowel function	1.7 (1.12)	1 (0.68)		1.62 (0.91)	
Prolapse symptoms	4.74 (2.01)	0.86 (1.07)	0.119 (*n* = 5)	0.67 (1.33)	0.022
Sexual function	2.56 (1.84)	0.34 (0.9)		0.67 (0.8)	
Total score	12.79 (5.4)	3.28 (3.38)	0.034 (*n* = 5)	4.2 (2.56)	0.081

Domains bladder, bowel, prolapse, sexual: 0 = best, 10 = worst

Total score: 0 = best, 40 = worst

*Friedman test with post hoc signed-rank test, *n* = number of included patients for analysis without imputation

Nine patients stated that they were satisfied with the operation.

## Discussion

Hornemann et al. first reported a laparoscopic apical prolapse repair using a semitendinosus tendon autograft. They confirmed its safety and efficacy in a multicenter study of 113 patients with a 6-month follow-up [5, 6]. We modified this technique to the robotic-assisted approach. Adding a straight needle temporarily to the tendon is a helpful step for tunneling the tendon through the cervix. Additionally, the robotic approach was very suitable for the different key steps of the procedure, particularly the nerve-sparing dissection and slitting of the anterior longitudinal ligament, the placement of the tendon through the cervix, the peritoneal tunnel, and through the anterior longitudinal ligament, and the attachment of the ends of the tendon by a side-to-side sling suture. Our results underline the success rate, showing that the robotic approach is feasible, especially in obese patients and those with intra-abdominal adhesions.

We also included the GPFQ, showing that robotic-assisted SCP not only reduces POP-Q point C but also has a positive impact on the patient’s quality of life.

Besides the semitendinosus tendon, there are other surgical approaches using biological grafts. Despite similar success rates, sacrocolpopexy using fascia of the rectus abdominis muscle or fascia lata has a complication rate of up to 41%, making implementation challenging [8]. The PROSPECT trial found no benefit of polypropylene mesh inlays or biological xenografts in the first 6 years postoperatively [9].

As a strength of our pilot study, we showed the implementation of a new surgical technique, including a mid-term follow-up of 12 months, with subjective and objective outcome parameters. However, limitations include the retrospective design and small number of patients, underlining that further multicenter, prospective studies are necessary to verify our results.

## Conclusion

This pilot study shows the feasibility of a modified robotic-assisted apical organ prolapse repair using the semitendinosus tendon, preparing for further prospective multicenter studies.

## Supplementary Information

Below is the link to the electronic supplementary material.

Supplementary file1 (MP4 67260 KB)

## Data Availability

The dataset and materials used in this study are available from the corresponding author upon reasonable request.
